# Tracking, Synthesizing, and Sharing Global *Batrachochytrium* Data at AmphibianDisease.org

**DOI:** 10.3389/fvets.2021.728232

**Published:** 2021-10-04

**Authors:** Michelle S. Koo, Vance T. Vredenburg, John B. Deck, Deanna H. Olson, Kathryn L. Ronnenberg, David B. Wake

**Affiliations:** ^1^Museum of Vertebrate Zoology, University of California, Berkeley, Berkeley, CA, United States; ^2^Department of Biology, San Francisco State University, San Francisco, CA, United States; ^3^Berkeley Natural History Museums, University of California, Berkeley, Berkeley, CA, United States; ^4^US Department of Agriculture, Forest Service, Pacific Northwest Research Station, Corvallis, OR, United States

**Keywords:** DarwinCore standards, amphibia, chytridiomycosis, MIxS standards, *Bd*, biodiversity informatics infrastructure, *Bsal*

## Abstract

Emerging infectious diseases have been especially devastating to amphibians, the most endangered class of vertebrates. For amphibians, the greatest disease threat is chytridiomycosis, caused by one of two chytridiomycete fungal pathogens *Batrachochytrium dendrobatidis (Bd)* and *Batrachochytrium salamandrivorans* (*Bsal*). Research over the last two decades has shown that susceptibility to this disease varies greatly with respect to a suite of host and pathogen factors such as phylogeny, geography (including abiotic factors), host community composition, and historical exposure to pathogens; yet, despite a growing body of research, a comprehensive understanding of global chytridiomycosis incidence remains elusive. In a large collaborative effort, *Bd*-Maps was launched in 2007 to increase multidisciplinary investigations and understanding using compiled global *Bd* occurrence data (*Bsal* was not discovered until 2013). As its database functions aged and became unsustainable, we sought to address critical needs utilizing new technologies to meet the challenges of aggregating data to facilitate research on both *Bd* and *Bsal*. Here, we introduce an advanced central online repository to archive, aggregate, and share *Bd* and *Bsal* data collected from around the world. The Amphibian Disease Portal (https://amphibiandisease.org) addresses several critical community needs while also helping to build basic biological knowledge of chytridiomycosis. This portal could be useful for other amphibian diseases and could also be replicated for uses with other wildlife diseases. We show how the Amphibian Disease Portal provides: (1) a new repository for the legacy *Bd-*Maps data; (2) a repository for sample-level data to archive datasets and host published data with permanent DOIs; (3) a flexible framework to adapt to advances in field, laboratory, and informatics technologies; and (4) a global aggregation of *Bd* and *Bsal* infection data to enable and accelerate research and conservation. The new framework for this project is built using biodiversity informatics best practices and metadata standards to ensure scientific reproducibility and linkages across other biological and biodiversity repositories.

## Introduction

The amphibian vertebrate lineage evolved over 360 million years ago, and has survived multiple mass extinction events, yet today amphibians are the most endangered class of vertebrates and may be harbingers of a new sixth mass-extinction event (1). Emerging infectious diseases have been especially devastating to amphibians (1–3). Chytridiomycosis is a potentially lethal amphibian skin disease caused by one of two chytridiomycete fungal pathogens, *Batrachochytrium dendrobatidis* (*Bd)* and *Batrachochytrium salamandrivorans (Bsal*). *Bd* chytridiomycosis, was first discovered over two decades ago (4–6), and later in 2013, *Bsal* chytridiomycosis was detected (7). A growing body of research has shown that amphibian susceptibility to these diseases varies phylogenetically, geographically, and is influenced by synergisms with abiotic and biotic factors (8, 9). However, a comprehensive understanding of the lethal, sublethal, and benign effects of these fungal pathogens and their long-term effects on vertebrates in class Amphibia is still incomplete. Chytridiomycosis has raised alarms and the World Organization of Animal Health (OIE) has listed *Bd* and *Bsal* as reportable pathogens (10) on the global stage. *Bd*, which includes multiple genetic lineages (11, 12), has spread across continents likely by human actions, and in some regions it has invaded naive host populations causing epizootics (epidemics in wildlife) that affect hundreds of species (13–15). The discovery of chytridiomycosis and the documentation of its impacts on amphibians have fundamentally altered the way scientists view emerging infectious diseases, their contributions to global biodiversity losses, and biodiversity conservation approaches to emerging disease threats (3, 8, 16).

We are facing a rapidly changing scientific knowledge landscape for amphibian emerging infectious diseases in the 21^st^ Century (7), which has increased the challenges for reporting and tracking advances (6). Indeed, the relatively recent discovery of the *Bsal* pathogen (7) has shown that we must be nimble in our approach to data management and analysis and adapt to new diseases and new technologies. Can we predict the data management needs of the next emerging disease that will infect amphibians or other wildlife species? What we do know is that sharing data and responding rapidly is essential to disease mitigation. As a scientific community we can heed lessons learned from our collective experience in *Bd* research for the last two decades. For example, we know that *Bd* can devastate entire amphibian populations (13) and entire amphibian communities quickly (14, 17). In outbreaks in Panama, 50% of local amphibian species were extirpated (17), and in Peru, 40% of species were extirpated (14). The advent of the discovery of *Bsal* motivated the formation of the North American *Bsal* Task Force (18), which included representatives from governmental, academic, and advocacy organizations in a broad coalition across the USA, Canada, and Mexico. Two of us (MK, DO) lead the *Bsal* Task Force Data Management Working Group. Our *Bsal* Task Force discussions clearly showed consensus for coordinated efforts in planning for when, not if, *Bsal* would eventually be detected in North America (19, 20). All parties agreed it is essential to track, archive, and quickly share sampling efforts for *Bsal*.

The first effort to compile *Batrachochytrium* occurrences for online access and mapping, beginning in 2007, resulted from a collaboration between the USDA Forest Service and Imperial College London known as *Bd*-Maps (21). The chytridiomycosis research community soon turned to *Bd*-Maps as the main source for compiled global data on *Bd* (21). This has been a labor-intensive aggregation of global *Bd* sampling efforts including >33,000 data records at >14,000 unique site coordinates to date (22). Based on both the *Bd*-Maps data and unique records recently summarized by Castro Monzon et al. (23), *Bd* has been detected in 1,375 of 2,525 (55%) species (composed of 88% of frog families, 100% of salamander families, and 70% of caecilian families) and 93 of 134 (69%) countries sampled to date [see (22) for details]. Over time, the labor-intensive methodology and unfunded infrastructure of the *Bd*-Maps became unsustainable. A new urgency arose when *Bsal* was discovered (7), and we embraced the challenge of how best to share data as quickly as it was produced and verified. Aggregating disease data can immediately address fundamental questions about where sampling effort has been applied, where disease occurrences are documented, and which species are affected, in addition to identifying active researchers to facilitate collaboration. In particular, it has become increasingly important to not only document known occurrences of *Bd* and *Bsal*, but also known instances of negative data (i.e., samples tested for *Bd* and/or *Bsal* that did not find evidence of infection), which by themselves may not be suitable for publication in many peer-reviewed journals. These data are critically important, however, in predictive distribution modeling [e.g., (22, 24)], and in examining host species traits such as phylogeny, habitat use, or behavior that may explain host infection or disease susceptibility [e.g., (25, 26)], and help understand the synergisms of co-occurring chytrid fungi [e.g., (27)]. If samples have linked genetic and genomic data, pathogenic fungal migration and evolution can be examined, revealing new insights on virulence, novel introductions, and origins of pathogens using phylodynamics [e.g., (11, 28–30)]. In addition, negative data can help describe the timing of pathogen invasion, which can also help in our understanding of present-day dynamics (31).

We introduce here an open-access repository and archive for *Batrachochytrium* data called the Amphibian Disease Portal (https://amphibiandisease.org) that addresses two urgent needs: (1) to create a sustainable, modernized repository to aggregate and rapidly share data on the fungal pathogens of amphibians *Bd* and *Bsal*; and (2) to upgrade and migrate the *Bd*-Maps datasets to a new repository that can continue to grow.

## Methods and Materials

In the creation of this online resource, we considered a broad range of users, facilitated by discussions with the *Bsal* Task Force, the AmphibiaWeb steering committee, and members of natural history museums and other institutions that provide biodiversity informatics data to users online. When planning how to store, structure, and share the data we considered the needs of conservation biologists, disease ecologists, evolutionary biologists, resource managers, and many others. We aimed to follow several principles to achieve these goals. The Amphibian Disease Portal was developed to: (1) prioritize structured data, data quality, and online accessibility, maximizing its usefulness and accessibility with modern web technologies and standards; (2) offer tangible benefits to users who are contributing their data (for the purposes of this paper, we refer to researchers who submit data as “contributors,” and those who access data for summaries or downloading for analysis as “portal users”); (3) be sustainable and cost-efficient for both maintenance and users (contributors and portal users will not be charged); and (4) support reproducible and replicable (i.e., repeatable methods with the same or comparable data, respectively, to produce the same results) data-driven science.

**Figure 1:**
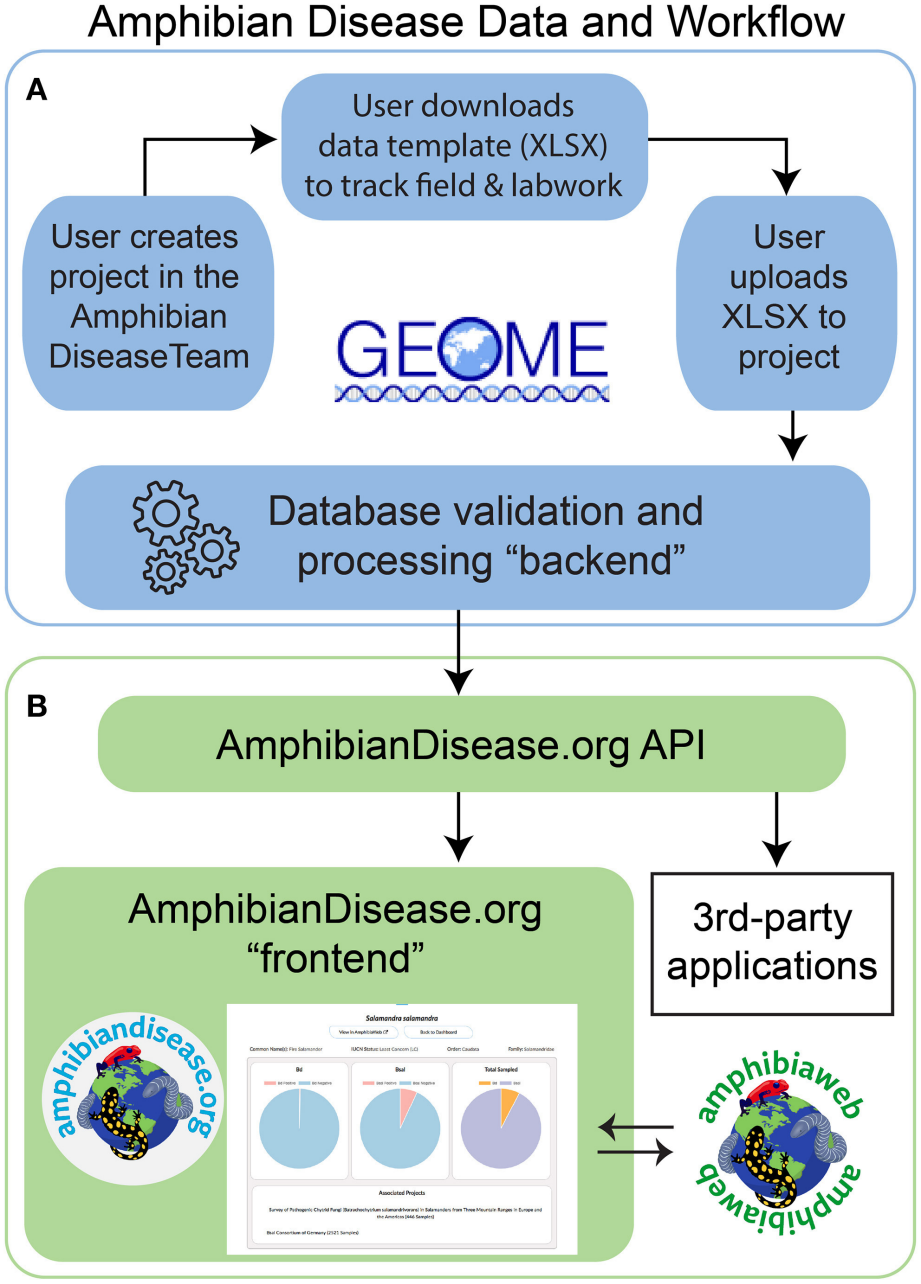
Amphibian Disease Portal data and work flow. **(A)** Users begin the process of contributing data by creating a project in Geome under the Amphibian Disease Team; user can download a data template for field and laboratory work. Once data gathering is complete, user uploads to the project for processing at Geome, and data are now available via the AmphibianDisease API. **(B)** The “frontend” includes the website AmphibianDisease.org where aggregated data can be graphed, mapped, queried, and downloaded. The AmphibianDisease API allows data to be accessible by any third-party application that can integrate web services like R Statistics. AmphibiaWeb site reciprocally links to Amphibian Disease species charts when data are available for a given species.

We initiated development in 2015, and the final architecture for the portal is described here. The overall architecture can be divided as a database “backend” and a user interface “frontend.” Many of our core goals align with an established metadata repository, the Genomic Observatories Metadatabase or GEOME (32), especially with respect to biological data management and structured, internet-accessible data. The GEOME repository offers a suite of user-friendly tools to manage and aggregate standardized biological sample data with its derivative genetic data, including associated geospatial, diagnostic, and publication context data [see [32] for core architecture]. We adopted the GEOME platform as the database and validation service for the ADP's “backend.” To meet the needs of research access and data access, we developed a dedicated “frontend,” a user-friendly website where all users can interact, visualize, and discover data. In addition, we created a middleware application programming interface (API) that communicates with the GEOME “backend.” Together, they comprise of the frontend, API, and backend components forming the Amphibian Disease Portal (Figure 1); all code is open-source and available on Github in two code repositories licensed as GNU General Public License (33), one for its frontend website (https://github.com/BNHM/AmphibiaWebDiseasePortal) and one for the API (https://github.com/BNHM/AmphibiaWebDiseasePortalAPI). We describe how we address and meet the principles and goals of the project with respect to its infrastructure.

The Amphibian Disease Portal has four interwoven Goals. (1) A key aim of the repository is to provide structured data, data quality, and online accessibility. GEOME is founded on a principle of providing interlinked and machine-readable data over the Internet, while drawing on standardized vocabularies from the scientific community. We built the repository with a focus on biological samples, and recognized the need to integrate data that may be housed in separate databases, especially gene-sequence data, which is critical in furthering our understanding of *Bd* and *Bsal* disease dynamics utilizing the field of phylodynamics (30). To achieve this goal, the portal uses DarwinCore (34) and MIxS (35) metadata standards, now common among biodiversity data repositories. The GEOME validation process checks data requirements such as appropriate diagnostic fields for *Bd* and *Bsal*, compliance with the AmphibiaWeb taxonomy (36), and controlled vocabularies for country, disease type, sample type, and basis of record. (Examples of controlled vocabularies are included in Table 1 and are lists of terms from which users can choose). Datasets with the DarwinCore fields of institutionID, collectionID, and catalogNumber constitute a global unique identifier for a cataloged specimen in a natural history museum collection, and may be linked to a biodiversity aggregator (e.g., Global Biodiversity Information Facility, GBIF); likewise, contributors may provide a Uniform Resource Identifier (or URI, e.g., a unique web address) for the field “associatedSequences” to link to external genetic repositories (i.e., National Center for Biotechnology Information, NCBI), for genomic Sequence Read Archive (SRA) or other molecular data. Contributors can choose to submit data directly to SRA through GEOME using its “FASTQ” module, in which case the sample is automatically linked between GEOME and SRA using the material Sample Identifier.

**Table 1:** Comparison of terms in legacy and current database.

**Bd-Maps (legacy terms)**	**Amphibian disease (DarwinCore compliant terms)**
• Record Number	• MaterialSampleID (unique identifier)
• SpecimenID	• otherCatalogNumber
• StartDay, StartMonth, StartYear, EndDay, EndMonth, EndYear	• dayCollected, monthCollected, yearCollected required For range of dates, these are noted in occurrenceRemarks
**Location** – (these generally had a DarwinCore counterpart)	
• Continent, Country, Region	• continentOcean, country, state_province
• Location	• locality
• Location Number	• locationID
• Latitude, Longitude	• decimalLatitiude, decimalLongitude
• Elevation (in meters)	• minimum ElevationInMeters, maximum ElevationInMeters
• CoordinateSource	• georeferenceProtocol (method and sources for determining coordinates)
• CoordinateAccuracy (free text)	• coordinateUncertaintyInMeters • georeferenceRemarks (derivation of coordinates, assumptions, and notes if coordinates are centroid-based)
• Order, Family, Genus, Species	• Order, Family, Genus, Species (validated using AmphibiaWeb taxonomy; synonyms applied)
• Species cf/aff/kl, Synonyms	• taxonRemarks (for additional specific epithet notes or remarks)
• WildCapt	• organismRemarks Controlled vocabulary of “wild,” “captive”
• MusSpec	• basisOfRecord Controlled vocabulary of “PreservedSpecimen” “LivingSpecimen,” “MaterialSample” etc. The term “Event” is used to distinguish Bd-Maps project data
• Test P/N/Q or U	• diseaseDetected Controlled vocabulary of “TRUE,” “FALSE”
**Bd Sampling Fields**	
Fields are tallies of lab results per location	
• BdDet – number of amphibians that had a positive Bd test	To map “BdDet”: • diseaseTestedPositiveCount “BdNeg” is not mapped.
• BdNeg - number of amphibians that had a negative Bd test	To map “Tested”: • individualCount
• Tested - number of amphibians that were tested for Bd	To map “Test Positive/Negative”: • diseaseDetected
• Test Positive/Negative – binary of P or N to facilitate filtering	Allowed values of “TRUE,” “FALSE”
**Site-level Summary** Fields are used for spatial metadata- analysis [see Olson et al. (22)], and are tallies for locations that share a locationNumber	Given the specific methodology used to compile these counts, these fields are concatenated to be included as a record note, with reference to Olson et al.
• Site Detected/Not Detected (D/ND) – tally of records for positive and negative samples	• eventRemarks
• Species with Detection – number of species with positive Bd detection	
• Species Tested – number of species tested	
• LifeStage, Sex	• lifeStage • SexBoth are controlled vocabulary fields
• Method	• testMethod
Free text for diagnostic test method	Controlled vocabulary for diagnostic test method; if more than one, note added to measurementRemarks
• Publication (also general provenance field if from unpublished source)	Concatenated into: • associatedReferences
• Data Source Type	
• Contact	• principalInvestigator
• Lab	• diagnosticLab
Other observations	If binary,
• Morbidity, mortality	• FatalAllowed values of “TRUE,” “FALSE” If free text, then concatenated into: • occurrenceRemarks

(2) We prioritized tangible benefits to the user to incentivize the contributions of researchers to the repository. Therefore, we considered the needs of the contributor foremost, including how and when to make a dataset publicly accessible. Many datasets are created for publication, and may have pre-publication restrictions (e.g., a graduate student's dissertation); hence, contributors can make newly uploaded datasets private and thus not searchable or accessible until a later date (e.g., when the study is published).

Even before uploading data, contributors may benefit from project planning and data-management tools provided by the portal. In GEOME, the Amphibian Disease Portal is identified as a “Team,” and all *Bd* and *Bsal* projects that are part of the team adhered to the database rules of the portal. These projects share not only database rules for *Bd* and *Bsal* samples but customized and customizable data templates. Contributors are encouraged to download and use these templates in Microsoft Excel format (XLSX) for data collection in the field and lab (See Supplementary Material for example templates). Instructions are included in each template file where fields and controlled vocabulary are defined and are clearly labeled. Once completed, the same file can be used to upload directly to the database without complicated formatting requirements. The portal has a minimum set of required fields and many optional fields from which users can customize their own template. Currently the Amphibian Disease Portal provides two different template configurations to any participating project: one designed for samples from museum specimens, and another addressing catch-and-release specimens from the field. We will continue to add new templates as new use cases arise, such as one for environmental samples and for laboratory or captive specimens. Along with an online guideline and instructions, we aimed to make data management easier during the data-collection phase.

Closing the cycle of a project for contributors is usually the publication step; journals are increasingly requiring authors to provide their data in an accessible archive. Data uploaded to the portal are provided with archival resource keys (ARK), a form of digital object identifier (DOI), generated by GEOME using California Digital Library's EZID service (37), and have satisfied data access requirements for journals to ensure that the data are citable, accessible, and credited.

(3) We designed the portal for flexibility, accessibility, and sustainability. By using representational state transfer (REST) web services (or the application programming interface, API), we display database-supplied data in easy-to-use formats, which appear on the Amphibian Disease Portal website via open access JavaScript libraries. Having data accessible through web services also allows third-party applications (e.g., R Statistics software) and other programming tools (e.g., Python) access to data without requiring secured database access. The portal's programming interface scripts are written in Python and allow for specialized processing such as matching taxonomy and synonyms from the AmphibiaWeb database. Using web services or APIs for the website has other development advantages, as well. We can deploy web developers as they are available from other projects for maintenance or let multiple developers work on new features for the site without relying solely on a database administrator, who has different skills and more strict access requirements. Thus, the architecture of the Amphibian Disease Portal allows for nimble management and enhancements as funding and needs change, providing a more sustainable project.

(4) Lastly, we aimed to support reproducible and replicable data-driven science. Reproducibility in science, under which independent researchers can repeat study results, requires access to the original sample data, yet published papers rarely contain those data and instead provide summarized data. Often the burden of providing the original data falls on scientists who have neither the time nor capacity to adequately store and retrieve data on request [e.g., (38)]. Replication in science, when applying comparable datasets to methods to test outcomes, requires access to datasets collated in a comparable manner, which also requires access to the original data. Submitting data to discipline-specific repositories compliant with well-known metadata standards, such as this portal, will reduce the future burden on the scientists who produced the data, and will provide aggregated data for re-use in potentially novel studies.

## Results

The outcome of this needs-based effort is the Amphibian Disease Portal, an online site with a user-friendly interface created in partnership with the GEOME repository. We established a user workflow that is easy to follow, cyber-secure, and makes data discoverable and accessible in a single site (Figure 1). Data and workflow include these steps: (1) registered contributors initiate projects based on their study and can use customized data templates in MS Excel format for project management (the same template, when completed after field and lab work is done, can be uploaded to their project); (2) uploaded data are validated against the portal's database rules (“backend”), which are defined in the template (feedback during this process is designed to help contributors correct and successfully load their data); (3) when the data are made public by the contributor, either immediately on upload or subsequent to publication, they will then be harvested and processed by the portal's web service; and 4) the data from public projects become output to the Amphibian Disease Portal (“frontend”) and other third-party applications (e.g., R program analyses).

**Figure 2:**
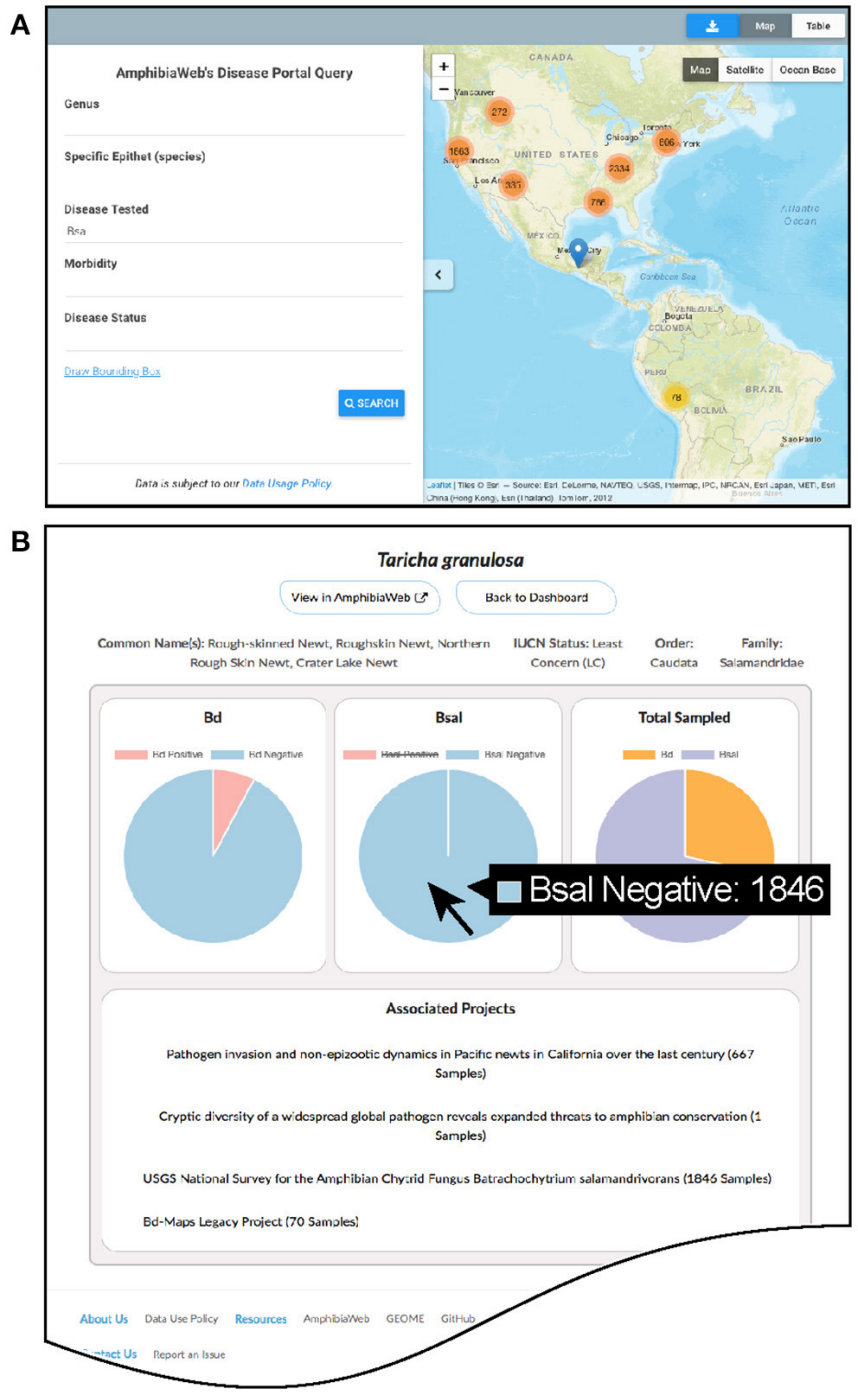
Examples of the Amphibian Disease portal's user-friendly pages. **(A)** Basic query and interactive mapping interface showing zoom-dependent clustering of data points with counts. **(B)** Dynamic graphing page for species showing proportion and counts of *Batrachochytrium dendrobatidis* (*Bd*) and *B. salamandrivorans* (*Bsal*) data with relevant links to projects (example species *Taricha granulosa*). Hovering over pie chart will display the specific counts of samples. All graphs in the Data Dashboard section of the website are enabled by the Amphibian Disease web services.

The main website (https://amphibiandisease.org) is comprised of: (1) a basic map-based query interface allowing for filtered or spatial searches and mapping; (2) a dashboard of summary statistics and dynamic charts by country and by species; (3) search interfaces for projects, species, and datasets; (4) various other information pages such as how to contribute data and a blog (Figure 2). Each species in the portal has a dedicated dashboard page to display aggregated *Bd* and *Bsal* samples with links to contributing projects. Graphs of species data (Figure 2B) are dynamic data-driven and reciprocally linked by URL to the respective species account page in AmphibiaWeb (https://amphibiaweb.org). The scripts enabling these reciprocal links are adaptable to other external websites as well.

With respect to the integration of the legacy Bd-maps dataset into the portal, our challenge in modernizing and migrating the *Bd*-Maps data is to overcome the differences in their original conception and data structure. Instead of a biological samples-based approach, *Bd*-Maps database compiles locality-based summaries by species as reported in diverse ways in the literature. Entries generally were not submitted by authors but compiled from literature queries or sent to the data manager as unpublished observations (21, 22). We elucidate the transition with an important migration step: matching all *Bd*-Maps fields to the DarwinCore standard equivalent used in the Amphibian Disease Portal. Table 1 compares the two respective metadata schemas.

Fields for date, taxonomy, and geography such as latitude and longitude, for example, were relatively straightforward to map. Three *Bd*-Maps fields that tally counts of total positive *Bd* samples (“BdDet”), total samples (“Tested”) and total number of samples with low zoospore levels which made them questionable (“BdQues” or “BdUnc”) required different accommodations, and are important to *Bd*-Maps based analyses [e.g., (21, 22)]. These fields were dependent on the “Location Number” field, which is matched to the portal field “locationID.” To make these data more usable in future studies, “BdDet” is mapped to a new field “diseaseTestedPositiveCount” and “Tested” is mapped to “individualCount.” Together they can be used to estimate prevalence (“diseaseTestedPositiveCount”/“individualCount”) for any given spatial aggregation or help recreate *Bd*-Maps by summarizing results for all records sharing a given “locationID.” Many of the other *Bd*-Maps data, especially those specialized for previous analysis are not abandoned but shifted to remarks fields. The Amphibian Disease Portal field “occurrenceRemarks” contains observations on morbidity and mortality; if mortality was noted, the portal field “fatal” was marked as “TRUE.” Details on the source and derivation for a *Bd*-Maps locality are captured in “georeferenceProtocol” and “georeferenceRemarks.” Other *Bd*-Maps notes on data sources (e.g., museum vs. field) or record type (species records as opposed to full records; 22) are concatenated into “occurrenceRemarks.” Finally, meta-analysis observations on number of *Bd* positive and negative species at a location are compiled into “eventRemarks” such that each record indicates whether a site has the disease Detected (D) or Not Detected (ND), number of species with positive *Bd* detection and the total number of species tested, separated by pipes for subsequent parsing by users; for example, “D | 2 | 4” indicates “*Bd* Detected, 2 positive out of 4 tested”. (For details of the Bd-Maps legacy database fields, refer to Appendix S1 of 22.)

**Figure 3:**
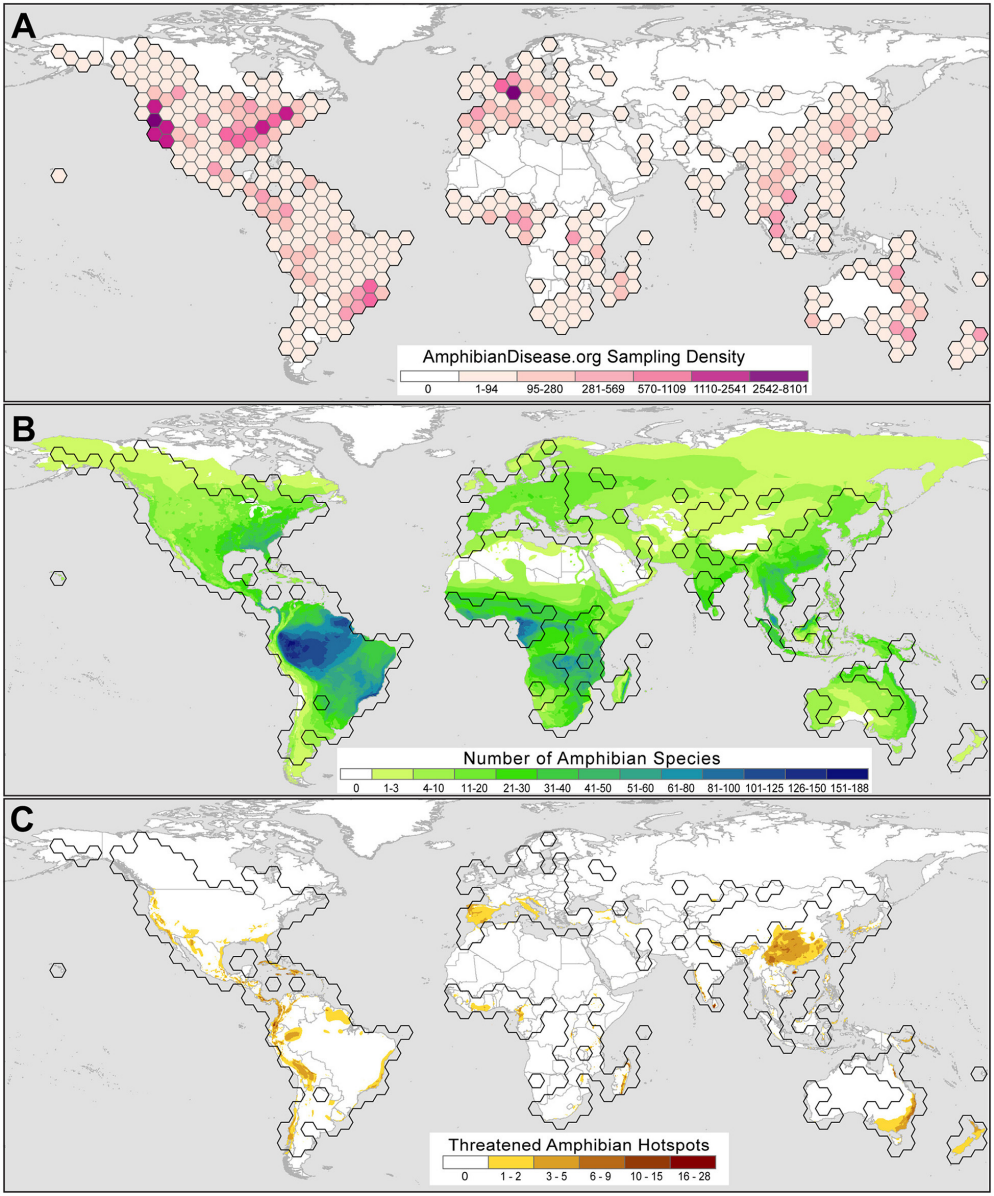
Amphibian Disease sampling point data for the chytrid fungal pathogens *Batrachochytrium dendrobatidis* (*Bd*) and *B. salamandrivorans* (*Bsal*) summarized by 5-degree latitude and longitude bins as of June 2021. **(A)** *Bd* and *Bsal* sampling is worldwide and reflects prominent sampling efforts for *Bsal* in the USA by the US Geological Survey (39) and in Germany by the *Bsal* Consortium Germany (42). Sampling strength is classified using natural breaks with Jenks optimization where darker colors denote greater numbers of samples per bin. **(B)** *Bd* and *Bsal* global sampling with respect to amphibian species alpha-diversity, where darker gradient denotes greater number of amphibian species. **(C)** *Bd* and *Bsal* global sampling with respect to threatened amphibian species, where darker gradient denotes greater number of threatened amphibian species. Range map sources: AmphibiaWeb (2021; https://amphibiaweb.org) and IUCN Red List (2021; https://www.iucnredlist.org). Threatened species status: IUCN Red List (IUCN 2021, accessed June 10, 2021).

Although usage, interest, and records in the portal are in their early growth stages, we observe trends from early user submissions, which may reflect broader research priorities and reveal useful patterns and gaps in effort. A total of 62,045 samples from 2,760 taxa and 128 countries are shown in 5-degree bins in Figure 3, including 34,267 records from the legacy *Bd*-Maps database (22) and excluding private datasets. Of these initial samples, about a quarter (26%) of the samples are derived from voucher specimens deposited in natural history museum collections, whereas the majority of records come from field surveillance and sampling of live amphibians globally. The repository includes the results from the first US-wide survey for *Bsal* (from May 2014 through August 2017) conducted by the US Geological Survey (39); more than 11,000 amphibians from reserves across the US were tested for *Bsa*l and all were *Bsal*-negative, providing a valuable baseline for future *Bsal* monitoring in North America. Lastly, one contributor has submitted pathogen sampling data from an ongoing monitoring program that uses environmental DNA (eDNA) analyses, in which organismal DNA either from shed skin cells or directly from microbes is found in terrestrial or aquatic habitats [e.g., (40, 41)]. The portal is flexible in its structure to accommodate these types of samples while adhering to metadata standards. The dynamic displays will be adapted to differentiate these records, which undoubtedly will grow in usage especially as a means of early detection.

## Discussion

The great amphibian epizootic, caused by *Bd* and *Bsal*, is on track to becoming a significant factor in an unfolding mass extinction event as extensive as the previous five recorded in Earth's geologic history (1, 43). Unlike previous extinction events, however, this one is unusual in that it is occurring over a significantly shorter time period (43), and at least for amphibians, disease is a major factor (1, 9, 23). Globally, amphibian chytridiomycosis has changed the way that we think about and understand wildlife disease (8, 44). For example, when amphibians were first reported to be mysteriously declining and disappearing in the late 1970s and early 1980s in Australia, Central America, and western North America (45), few imagined so much of the decline could be driven by epizootics caused by a single pathogen [(46, 47) but see (48)]. At the time, our relatively poor understanding of the biology of Chytridiomycete fungi and a general perception that a fungal pathogen could not drive populations or species to extinction, likely contributed to the fact that it took nearly 20 years to identify *Bd*, discovered in 1998 (4) and described in 1999 (5), as a proximal cause of amphibian declines and extinctions.

The study of chytridiomycosis has led to significant breakthroughs in our understanding of the factors that can lead to outbreaks (epizootics) and population collapse, or non-fatal pathogen infections of hosts (9, 49). Yet, our ability to predict infection outcomes has been hampered because of the great variation in the timing of when this pathogen invaded different continents, the array of species traits in the numerous amphibian hosts, the biotic conditions under which it can infect amphibians, and the multiple genetic lineages differing significantly in virulence (8, 11, 12). Studies have described widely divergent host-pathogen dynamics in this system. Some characterize *Bd* host dynamics as stable enzootics where hosts do not succumb (50), whereas in other settings, even though they may consist of the same host species, living in the same nearby environment, *Bd*-host dynamics are characterized as epizootic, and hosts suffer mass die-offs (13). It has become evident that our ability to understand this disease and predict the outcome of infection relies on many complex variables including but not limited to the timing of pathogen invasion (31). Thus, understanding present-day disease dynamics may require describing the past. The Amphibian Disease Portal contains critical archival *Bd* data that provide unique historic insights. Cheng et al. (51) demonstrated that amphibian museum specimens can be successfully tested for the presence of *Bd*, and thus opened the door to studies of *Bd* temporal dynamics over long timescales (over a century). Other studies followed using museum specimens to help describe *Bd* distributions in the past (31, 52–65), revealing the unique value of museum specimen collections to disease ecology, and the importance of negative data. This realization has set next priorities to develop a voucher specimen lookup service, which will facilitate incorporation of collection records in data templates and increase data quality.

Currently, the data from the growing base of portal users show that global amphibian disease sampling efforts are uneven (Figure 3A); baseline *Bd* and *Bsal* sample data cover some amphibian hotspots but only lightly sample others (Figure 3B). For example, the Amazon basin is the most species-rich region for amphibians and yet sampling is sparse. Likewise, there are few samples from the range of the Western Ghats (India), which has high beta-diversity. However, a baseline of *Bd* and *Bsal* data for the Appalachian region in southeastern North America is forming, which is a global hotspot for salamander diversity (24, 66). The portal can visualize *Bd* and *Bsal* sampling in areas where threatened amphibian species occur [species with IUCN Red List status of Critically Endangered, Endangered or Vulnerable, (67)] and may help prioritize monitoring and surveillance efforts for the *Bd* and *Bsal* pathogens (Figure 3C). Regions where an accumulation of threatened species are known and baseline *Bd* and *Bsal* data have been collected include Central America south through the Andean cordillera (S. America), regions in which studies have increased our understanding of chytrid disease dynamics [e.g., (13, 51, 68, 69)].

Outcomes of chytridiomycosis also are influenced by host species (70, 71), pathogen lineage (72, 73), host community (74, 75), host microbiome (49, 76–79), abiotic conditions (75, 80), and host and pathogen population genetics (11, 12, 72). This complex reality requires collaboration between research groups allowing for sharing and visualization of original, raw data (not summarized data), in ways that are not currently possible with the peer-review-based, publication-based science that is currently the norm. The Amphibian Disease Portal provides a platform to share data on pathogen lineage, host traits, and a suite of metadata associated with where the samples were collected (various abiotic and biotic factors) as well as other potential cofactors that may yet be identified.

Disease ecology, in particular the study of emerging infectious diseases, is decidedly hampered by the standard peer-reviewed science approach because negative data are not typically published. For emerging infectious diseases, knowing that a host population was negative for a pathogen before pathogen invasion and emergence is critically important. Researchers need to be able to share pre-publication data with trusted collaborators (password protected). In conservation emergencies, they should also be able to share data in a completely open format (when appropriate) in ways that many current peer-review-based, scientific publications do not allow. Knowing whether a host population is naïve to an invading pathogen completely alters the prediction of disease outcome (50). If hosts are naïve to a pathogen, they are predicted to be much more vulnerable to epizootic dynamics and host mortality (50) than if they have previous experience with the pathogen (50, 81). The Amphibian Disease Portal provides that missing scientific platform to archive host/pathogen data by providing unique and citable digital object identifiers (DOI), making them available for the benefit of science and for the conservation of species. The uploaded data in the portal must follow strict, yet simple, rules that ensure compatibility of data across studies. Researchers must upload specific details that do not vary across projects but are given the opportunity to provide additional data (e.g., host size and weight) that might prove to be important after further analysis. The portal also gives researchers the option of storing their data in a private, password-protected environment when necessary; however, if the situation is deemed an emergency, researchers can quickly make the data available to the public. With the creation of the Amphibian Disease Portal, we show that we can harness new technologies to increase collaboration and communication among scientists globally using a workflow that is simple, sustainable, and low-cost. We provide a place to rapidly access data not only from published papers, but also from researchers who are willing to share data not yet published. The Amphibian Disease Portal enables digital object identifier assignments for these data regardless of whether they are published in a journal and allows scientists to cite their data on a stable platform that is permanently accessible. Because the portal stores original data and not summarized data, researchers are able to access the raw sample data and potentially analyze them in novel ways. This is significant because it means that data will be more easily compiled and compared across different studies. For research that is global in scope, such as studies of emerging infectious diseases (e.g., the great amphibian panzootic), being able to access and share verified data across studies is essential. In addition, the portal provides a flexible framework that allows for new research results to be added quickly and efficiently. For example, new studies are showing that eDNA methods (82) can detect the presence of *Bd* in aquatic systems, and this could greatly increase sampling across geographic space and could help direct limited resources toward organismal surveys in key areas. Additionally, the portal can link to existing biodiversity datasets to integrate access to genomic/genetic raw sequence data that could facilitate research on lineages of *Bd* pathogens (11, 12, 83–85). Thus, the portal utilizes critical functions of modern biodiversity data repositories and promotes open science practices. The lessons of the past decades of emerging infectious disease studies in amphibians have focused our attention on a few focal pathogens with the recognition that we need to expand our temporal and genomic investigations. The Amphibian Disease Portal is designed to maximize compatibility, access, and value of data to understand the current disease dynamics and to predict and adapt to future disease threats.

## Data Availability Statement

Publicly available datasets were analyzed in this study. This data can be found at: https://amphibiandisease.org and https://geome-db.org/workbench/project-overview?projectId=291.

## Author Contributions

MK conceived, designed, led development, and writing of the study. VV conceived, consulted, and wrote the study. JD designed and wrote the study. DO, KR, and DW consulted and wrote the study. All authors approved the final version of this manuscript.

## Funding

Funding for initial development was provided by a USDA Forest Service Cooperative Agreement #15-CR-11261953-098 (University of California Berkeley) and a Belmont Forum Project NSF #1633948 to Vredenburg. The use of trade or firm names is for reader information and does not imply endorsement by the U.S. Department of Agriculture of any product or service.

## Conflict of Interest

The authors declare that the research was conducted in the absence of any commercial or financial relationships that could be construed as a potential conflict of interest.

## Publisher's Note

All claims expressed in this article are solely those of the authors and do not necessarily represent those of their affiliated organizations, or those of the publisher, the editors and the reviewers. Any product that may be evaluated in this article, or claim that may be made by its manufacturer, is not guaranteed or endorsed by the publisher.
